# Effects of Fermented and Unfermented Chinese Herbal Formulations on Laying Performance, Intestinal Health and Cecal Microbiota in Late-Phase Laying Hens

**DOI:** 10.3390/ani16213510

**Published:** 2026-11-06

**Authors:** Siqiang Liu, Yijie Wang, Miaoyang Ding, Yudian Zhao, Ruirui Jiang, Xiangtao Kang, Qiang Li, Yujie Gong, Yadong Tian

**Affiliations:** 1 College of Animal Science and Technology, Henan Agricultural University, Zhengzhou 450046, China; 2 Henan Province Engineering Research Center of Poultry Germplasm Resources and Feed Nutrition, Zhengzhou 450046, China; 3 College of Animal Science & Technology, Henan University of Animal Husbandry and Economy, Zhengzhou 450046, China

**Keywords:** Chinese herbal medicine, production performance, gut health, egg quality, aged laying hens

## Abstract

The intestinal structure and function of laying hens undergo age-related degeneration, which can impair nutrient absorption and reduce production performance. Chinese herbal additives have been investigated for their potential benefits in supporting intestinal health and physiological function. In this study, a compound seven-herb formula was evaluated for its potential to support intestinal health in late-phase laying hens. The study aimed to compare the effects of unfermented and fermented herbal formulations on laying performance, egg quality, and intestinal health in late-phase laying hens. A total of 540 hens were randomly assigned to three groups (six replicates per group, 30 hens per replicate) and fed either a basal diet (Control), a diet supplemented with Chinese herbal medicine (CHM), or a diet supplemented with fermented Chinese herbal medicine (FHM) for 12 consecutive weeks. During weeks 9–12, egg production rate was significantly higher in the CHM and FHM groups than in the control group (*p* < 0.05). Over the entire experimental period, average egg weight was significantly higher in the FHM group than in the control and CHM groups (*p* < 0.05). Both treatments also improved selected indices of intestinal morphology, including villus height and the villus height-to-crypt depth ratio (*p* < 0.05). Additionally, serum concentrations of immunoglobulin A (IgA), the immunoglobulin reported as IgG, and immunoglobulin M (IgM) were significantly increased, whereas intestinal concentrations of interleukin-1β (IL-1β), interleukin-8 (IL-8), and transforming growth factor-β (TGF-β) were reduced in both treatment groups (*p* < 0.05). Both CHM and FHM supplementation reduced serum malondialdehyde (MDA) concentrations and increased superoxide dismutase (SOD) activity, although serum glutathione peroxidase (GSH-Px) activity was decreased (*p* < 0.05). Furthermore, FHM altered the cecal microbial composition and increased the concentrations of acetate, butyrate, and isovalerate. In conclusion, dietary herbal supplementation, particularly in fermented form, may improve selected production-performance traits and intestinal health in aged laying hens.

## Simple Summary

Maintaining egg production and intestinal health becomes increasingly challenging as laying hens age. Herbal feed additives may offer a nutritional approach to supporting the health and productivity of hens during the later laying period. In this study, 540 laying hens were fed a standard diet or diets supplemented with unfermented or fermented Chinese herbal formulations for 12 weeks. Both herbal treatments increased egg production during the final four weeks of the trial, while the fermented formulation increased average egg weight over the entire experimental period. Dietary herbal supplementation was also associated with changes in intestinal structure, antioxidant status, immune-related parameters, and gut microbial composition. The fermented formulation additionally increased the concentrations of certain short-chain fatty acids in the cecum. These findings suggest that Chinese herbal formulations may have potential as functional feed additives to support laying performance and intestinal health in late-phase laying hens. However, further research is needed to determine the specific contribution of fermentation to these effects.

## 1. Introduction

Traditional Chinese medicine (TCM) contains a wide range of bioactive compounds and has been increasingly explored as a source of functional feed additives due to its potential antioxidant, immunomodulatory and gut health–promoting properties [1]. In the context of increasing restrictions on antibiotic growth promoters, phytogenic feed additives have received growing attention as sustainable alternatives, although their efficacy and practical application depend on appropriate standardization, formulation, and safety evaluation [2]. With the rapid advancement of the fermented feed industry, efforts have been made to integrate TCM with fermentation technology. This combination has been shown to enhance the bioavailability and pharmacological activity of herbal compounds, reduce toxicity, and even generate novel bioactive constituents [3]. At present, fermented herbal medicine has been successfully applied as a dietary supplement in a variety of economically important animals, including dairy cattle, weaned piglets, and aquatic species such as crucian carp [4,5,6]. In the poultry industry, fermented herbal medicine has been reported to improve growth performance in chicks [7], as well as enhance egg-laying performance and intestinal health in laying hens during peak production periods [8]. However, whether these beneficial effects extend to aged laying hens, which experience progressive deterioration of intestinal structure and function during the late laying period, remains unclear.

During the late laying period, hens may experience intestinal barrier impairment, immune dysfunction, and gut microbiota dysbiosis, all of which can negatively impact nutrient metabolism and overall health [9]. Recent studies have highlighted a strong association between intestinal health and age [10], commonly characterized by compromised intestinal barrier integrity [11,12], increased intestinal permeability, reduced resistance to pathogens [13], heightened inflammatory responses [12], decreased microbial diversity, and disrupted metabolic function [14,15]. These age-related changes in intestinal function may represent a critical factor contributing to the decline in production performance in aging laying hens, independent of reproductive decline.

Given the relationship between intestinal health and production performance, the formulation combined herbs traditionally used for kidney support with herbs used for gastrointestinal regulation. Several traditional Chinese herbal ingredients, including *Angelica sinensis*, *Astragalus membranaceus*, *Dipsacus asper*, *Cornus officinalis*, *Poria cocos*, *Citrus reticulata*, and *Glycyrrhiza uralensis*, were selected based on their traditional functions. Among these, *Angelica*, *Astragalus*, *Dipsacus*, and *Cornus* were included for their traditional tonic properties, while *Poria* (25%) and *Citrus* (20%) were incorporated at higher proportions to emphasize gastrointestinal health. *Glycyrrhiza* was included as a complementary ingredient to harmonize the formulation. These proportions were selected based on the intended functional emphasis of the formulation rather than through formal dose–response optimization. In addition, *Citrus reticulata* has been shown to promote gastric mucosal repair in rats [16]. *Poria cocos* and *Cornus officinalis* may contribute to the observed changes in intestinal health-related indicators in late-phase laying hens [17,18], while studies in murine models have also suggested potential effects on gastrointestinal motility and digestion [19]. These findings suggest that *Citrus*, *Poria*, and *Cornus* may also play a beneficial role in improving intestinal health in laying hens.

To date, however, research on the combined application of traditional tonic herbs and gut-regulating herbal ingredients in aged laying hens remains limited. Therefore, this study aimed to evaluate the effects of fermented Chinese herbal formulations prepared by solid-state fermentation on the production performance and intestinal health of aged laying hens.

## 2. Materials and Methods

### 2.1. Test Materials

The herbal ingredients were purchased from Huatuo International Chinese Medicine City, Kangmei (Bozhou), Anhui, China. Additional materials included wheat bran, corn meal, soybean meal, maifan stone, molasses, and *Bacillus subtilis* powder (100 billion CFU/g), a compound lactic acid bacteria powder (30 billion CFU/mL, Chinese trade name: YouKangSu), both supplied by Luoyang Oukebaike Biotechnology Co., Ltd. (Luoyang, Henan, China). The composite lactic acid bacteria powder contained *Enterococcus faecalis*, *Micrococcus lacticum* and *Bacillus coagulans*. Feeding experiments were conducted at Fengyuan Poultry Co., Ltd., Nanyang City, Henan Province, China. The experimental animals were Hy-Line Brown laying hens, and a commercial diet formulated for late-laying hens was used as the basal diet. Table 1 displayed the proportions of various herbal ingredients in the formula. All Chinese herbal materials were ground into powder before use, passed through a 40-mesh sieve, and weighed according to the proportions in Table 1 to prepare a mixture of the herbal powders. The fermentation substrate was prepared by mixing bran, corn meal, soybean meal, and maifan stone at a ratio of 16:11:8:5. The herbal powder mixture and carrier substrate were combined at a ratio of 5:2 (*w*/*w*) before fermentation. Molasses was added at one-quarter of the carrier substrate weight, giving an input mass ratio of herbal powder, carrier substrate, and molasses of 100:40:10. The molasses was dissolved in warm water at approximately 35 °C. The activated bacterial solution was prepared by adding 0.5 g of compound bacterial preparation and 0.04 g of *Bacillus subtilis* per kilogram of final fermented herbal preparation, stirring, and allowing it to stand for 30 min. The activated bacterial solution, herbal mixture, and substrate mixture were thoroughly mixed and adjusted to a moisture content of around 40% to obtain a wet solid material. Finally, the wet solid material was packed into fermentation bags with one-way exhaust valves (25 kg, 51 × 81 cm), the air was manually expelled, and the bags were then incubated at 30 °C for 7 d. The finished product was stored in a refrigerated room at 4 °C for future use.

### 2.2. Animals and Diets

A total of 540 healthy Hy-Line Brown laying hens at 433 days of age, with no significant difference in egg production rate (*p* > 0.05), were randomly divided into 3 experimental groups, with 6 replicates per group and 30 hens per replicate. The hens were housed in a three-tiered cage system (upper, middle, and lower tiers), with 10 cages per replicate and three hens per cage. The replicate was considered the experimental unit. The CHM group received 4.8 g of the unfermented herbal powder mixture per kilogram of diet, whereas the FHM group received 20 g of the composite fermented product per kilogram of diet. The latter contained the same seven-herb formulation together with carrier substrates, molasses, and microbial preparations. Following a one-week adaptation period, the control group was fed the basal diet, whereas the two treatment groups received the basal diet supplemented with 0.48% CHM or 2% FHM (as-fed basis), respectively, for 12 weeks. The 2% FHM inclusion level was selected based on a previous study in late-laying hens [20]. The proportions of the basal dietary ingredients were adjusted to provide comparable levels of metabolizable energy, crude protein, and other major nutrients among the three experimental diets. Throughout the experiment, all hens had ad libitum access to feed and water under a lighting schedule of 16 h light and 8 h darkness. Routine immunization and management procedures were followed according to the farm’s standard practices. Eggs were collected daily, and feed consumption was recorded weekly for each replicate. The calculated metabolizable energy and analyzed nutrient composition of the diets are presented in Table 2.

### 2.3. Sample Collection

At the end of the 12-week feeding trial, the laying hens were fasted for 12 h before blood and tissue sampling. Ten fresh eggs were randomly collected from each replicate (60 eggs per treatment) for egg quality assessment. The mean value of the 10 eggs within each replicate was used for statistical analysis (*n* = 6). One hen was randomly selected from each replicate for blood collection and was subsequently euthanized for tissue and cecal-content sampling (*n* = 6). All the blood samples were centrifuged at 3000 rpm for 15 min at 4 °C, and the upper layer of serum was aspirated and stored at −20 °C for further use. After euthanasia by cervical dislocation, liver, ovary and intestinal tissues were collected for further analysis. The duodenum, jejunum, and ileum were quickly removed, and a 2 cm segment from the middle portion of each intestine was taken. The intestinal segments were rinsed with sterile phosphate-buffered saline (PBS) and then fixed in 4% paraformaldehyde for morphological analysis. The cecal contents were collected for analysis of the gut microbiota.

### 2.4. Chemical Composition Analysis

Feed samples were collected before fermentation (0 d) and after 1, 2, 3, 4, and 7 days of fermentation. The populations of lactic acid bacteria, *Bacillus* spp., and yeasts in the fermented herbal feed were determined using plate-count methods as described by Woo et al. [21], with appropriate selective culture media used for each microbial group. The pH was measured using a pH meter (PHS-2F, Shanghai Instrumentation Scientific Instrument Co., Ltd., Shanghai, China) (Figure S1). Microbial counts were determined during fermentation, but the viability of individual inoculated microorganisms after refrigerated storage and at the time of feeding was not assessed. The proximate chemical composition of the herbal ingredients was determined using the Association of Official Analytical Chemists (AOAC) procedures [22], including crude protein, dry matter, ether extract, and crude ash. Acid detergent fiber (ADF) was determined according to the methods specified in standard NY/T1459-2022 [23] (Table S1). Neutral detergent fiber (NDF) was determined according to GB/T 20806-2022 [24] (Table S1).

### 2.5. Egg Production Performance and Egg Quality

Eggs were collected daily, and the number of eggs and total egg weight were recorded for each replicate. Feed intake was recorded weekly for each replicate by weighing the feed supplied and the feed remaining. Feed consumption was calculated as the difference between the amount supplied and the amount remaining. Average egg weight was calculated as total egg weight divided by the number of eggs. Average daily feed intake (ADFI) was calculated based on the total feed consumed and the corresponding number of bird-days, defined as the sum of the number of hens present on each day. Feed conversion ratio (FCR) was calculated as the ratio of total feed consumption to total egg weight produced within each replicate. Egg production rate, average egg weight, ADFI, and FCR were calculated for the periods of 1–4 weeks, 5–8 weeks, 9–12 weeks, and the entire experimental period. Ten fresh eggs from each replicate (60 eggs per treatment) were used to determine egg weight, albumen height, yolk color, Haugh unit, eggshell thickness, and eggshell strength. The mean value of the 10 eggs within each replicate was used as one experimental observation for statistical analysis (*n* = 6). Measurements were performed as described by Wang et al. [25], as follows. Eggshell color was measured using a portable colorimeter (CR-400, Beijing Kemeirunda Instrument Co., Ltd., Beijing, China), and the resulting color index was recorded according to the instrument specifications. Yolk color was measured using an egg quality analyzer (EMT-5200, Robotmation Co., Ltd., Tokyo, Japan) and expressed on a 1–15 scale based on the Roche Yolk Color Fan, with higher scores indicating darker yolk pigmentation. Egg length and width were measured using a vernier caliper, and the egg shape index was calculated as the ratio of egg length to egg width. The yolk was separated from the albumen and weighed using an electronic balance. The yolk proportion was calculated as the ratio of yolk weight to whole egg weight.

### 2.6. Determination of Antioxidant Indexes

The levels of malondialdehyde (MDA) and the activities of glutathione peroxidase (GSH-Px), superoxide dismutase (SOD) and catalase (CAT) in serum, liver and ovarian tissue were determined according to the instructions in the kit produced by Nanjing Jiengcheng Bioengineering Institute, Nanjing, China.

### 2.7. Intestinal Morphology

The fixed intestinal segments were subjected to tissue dehydration, clearing, and paraffin embedding to prepare paraffin sections. The tissue sections were stained with hematoxylin and eosin, and then observed using a digital slide scanner (MoticEasyScan Pro, Motic China Group Co., Ltd., Xiamen, China) and slide preview software (Motic DSAssistant Lite 1.0, Motic China Group Co., Ltd., Xiamen, China). The villus height (V), crypt depth (C), and villus-to-crypt ratio (V/C) were measured and calculated. For each intestinal segment, ten well-oriented villus–crypt units were measured per bird, and the mean value was used for statistical analysis.

### 2.8. Relative mRNA Expression Levels of Genes

Total RNA was isolated from the ileum tissue using TRIzol reagent (Vazyme, Nanjing, China) according to the manufacturer’s instructions. The concentration and integrity of the RNA were evaluated using a NanoDrop 2000 spectrophotometer (Thermo Fisher Scientific, Wilmington, DE, USA) and 1% agarose gel. The HiScript^®^ III 1st Strand cDNA Synthesis Kit (+gDNA wiper) (Vazyme, Nanjing, China) was used to synthesize cDNA by reverse transcription of RNA. The qRT-PCR reaction was prepared using the ChamQ Universal SYBR qPCR Master Mix (Vazyme, Nanjing, China) and was performed using a real-time fluorescence quantitative PCR instrument (LightCycler 96, RocheDiagnostics GmbH, Mannheim, Germany). The qRT-PCR primers were designed using Primer-BLAST(version 2.5.0)in National Center for Biotechnology Information (Table 3), and chicken β-actin gene was used as an internal control for gene expression normalization. The relative expression abundance of each target gene was calculated using the 2^−ΔΔCt^ method [26].

### 2.9. Serum Immunoglobulin Content and Inflammatory Factors in the Intestine

The intestinal tissue was mixed with sterile cold PBS in a ratio of weight to volume of 1:9. After thorough homogenization of the mixture, it was centrifuged at 2500 rpm for 20 min at 4 °C, and the supernatant was collected and reserved. Following the instructions provided by the manufacturer (Jiangsu Enzyme Immuno Industrial Co., Ltd., Yancheng, Jiangsu, China), specific enzyme-linked immunosorbent assay (ELISA) kits were used to determine the levels of immunoglobulin M (IgM), immunoglobulin G (IgG), and immunoglobulin A (IgA) in the serum, and the levels of Interleukin-1β (IL-1β), Interleukin-8 (IL-8), transforming growth factor-β (TGF-β), and tumor necrosis factor-alpha (TNF-α) in the intestinal tissue homogenate supernatant.

### 2.10. Cecal Microbiota Analysis by 16S rRNA Gene Sequencing

The method described in the study by Guo et al. [27] was used to perform 16S rRNA gene sequencing on the cecal contents of each replicate laying hen. This analysis was conducted by Majorbio Bio-Pharm Technology Co. Ltd. (Shanghai, China). The V3-V4 region of bacterial 16S rRNA genes was PCR-amplified using the universal primers 338F (5′-ACTCCTACGGGAGGCAGCAG-3′) and 806R (5′-GGACTACHVGGGTWTCTAAT-3′), followed by paired-end sequencing on an Illumina MiSeq PE300 platform (Illumina, Inc., San Diego, CA, USA). Raw sequences were quality-filtered using fastp (version 0.19.6) and merged using FLASH (version 1.2.11). The optimized sequences were clustered into operational taxonomic units (OTUs) at 97% similarity using UPARSE (version 11), and representative sequences were taxonomically classified against the SILVA 16S rRNA database (release 138) using the RDP Classifier (version 2.13) with a confidence threshold of 70%. OTU distributions were visualized using Venn diagrams, and beta diversity was evaluated by principal coordinate analysis (PCoA) based on Bray–Curtis distances. Microbial community composition was analyzed at the phylum and genus levels, and differences in taxonomic abundance among groups were assessed using the Kruskal–Wallis H test.

### 2.11. Determination of Short-Chain Fatty Acids in Cecal Contents

Cecal contents were weighed and homogenized with 0.5% phosphoric acid solution at a concentration of 10 mg/mL. The mixture was frozen, ground, and then subjected to ultrasonication for 10 min at 4 °C, followed by centrifugation at 13,000× *g* for 15 min. The resulting supernatant was collected and extracted with n-butanol. After a second round of ultrasonication and centrifugation under the same conditions, the final supernatant was obtained for analysis. Short-chain fatty acids (SCFAs) were identified and quantified using a gas chromatography–mass spectrometry (GC-MS) system (8890B–5977B GC/MSD, Agilent Technologies Inc., Santa Clara, CA, USA). Compound identification and peak integration were performed using MassHunter Quantitative Analysis software (version 10.0.707.0, Agilent Technologies). The concentrations of individual SCFAs were calculated based on external standard curves generated from known concentrations of reference standards.

### 2.12. Statistical Analysis

The data analysis was performed using IBM SPSS 24.0 (SPSS Inc., Chicago, IL, USA). One-way analysis of variance (ANOVA) was applied to assess differences among groups, followed by Tukey’s multiple comparison test to determine statistical significance. The replicate was considered the experimental unit. For egg quality traits, the 10 eggs sampled from each replicate were treated as subsamples, and their mean value was used for statistical analysis (*n* = 6). The data were presented as the mean and standard error of the mean (SEM). Differences were considered statistically significant at *p* < 0.05, whereas 0.05 ≤ *p* < 0.10 was interpreted as a statistical trend. Graphs of relative mRNA expression and SCFA concentrations were generated using GraphPad Prism 8.0.2 (GraphPad Software, San Diego, CA, USA).

## 3. Results

### 3.1. Laying Performance and Egg Quality

The impact of fermented Chinese herbs on egg production performance is presented in Table 4. Egg production rate did not differ among treatments during weeks 1–4 or 5–8 (*p* > 0.05). During weeks 9–12, egg production rate was significantly higher in the CHM and FHM groups than in the control group (*p* = 0.002). Average egg weight was higher in the FHM group than in the control group during weeks 1–4 (*p* = 0.035) and was higher in the FHM group than in both the control and CHM groups during weeks 5–8 (*p* = 0.009). During weeks 9–12, average egg weight was highest in the FHM group, followed by the CHM and control groups (*p* < 0.001). Over the entire experimental period, average egg weight was significantly higher in the FHM group than in the control and CHM groups (*p* = 0.001). ADFI was significantly lower in both the CHM and FHM groups than in the control group over the entire 12-week period (*p* = 0.004). FCR was lower in the FHM group than in the control group during weeks 1–4 (*p* = 0.045). The impact of different dietary treatments on egg quality in laying hens is presented in Table 5. Compared with the control group, both the CHM and FHM groups showed a tendency to improve eggshell strength. The yolk color of the FHM and control groups was significantly higher than that of the CHM group. Additionally, the yolk ratio in the CHM and FHM groups was significantly lower than that in the control group, while the Haugh unit of the CHM and FHM groups was significantly higher (*p* < 0.05).

### 3.2. Antioxidant Status

The effects of dietary treatments on the antioxidant parameters of laying hens are shown in Table 6. In serum, GSH-Px activity was significantly lower in both the CHM and FHM groups than in the control group (*p* < 0.001), with the CHM group exhibiting the lowest activity. In contrast, SOD activity was significantly increased in both treatment groups, and the highest value was observed in the FHM group (*p* = 0.002). Serum MDA concentration was significantly reduced in both CHM and FHM groups compared with the control (*p* = 0.002). CAT activity was significantly increased in the CHM group, whereas no significant difference was observed between the FHM and control groups (*p* = 0.004). In the liver, SOD activity was significantly enhanced in the CHM and FHM groups compared to the control (*p* < 0.001), with the FHM group showing the highest value. However, there were no significant differences in GSH-Px, CAT, or MDA levels among the groups (*p* > 0.05). In the ovary, GSH-Px activity was significantly higher in the CHM and FHM groups than in the control (*p* = 0.002). SOD activity was also significantly increased in the CHM group compared to the control, while the FHM group showed intermediate values (*p* = 0.008). MDA levels were significantly reduced in both CHM and FHM groups compared to the control group (*p* = 0.002). CAT activity was significantly elevated in the FHM group (*p* = 0.011), whereas the CHM group showed no significant difference from the control.

### 3.3. Intestinal Morphology

The effects of dietary treatments on intestinal morphology are presented in Table 7. In the duodenum, no significant differences were observed in villus height among the three groups (*p* = 0.774). However, crypt depth was significantly lower in both the CHM and FHM groups compared to the control (*p* = 0.033), with the FHM group exhibiting the shallowest crypts. The V/C ratio was significantly increased in the FHM group (*p* = 0.026), suggesting improved intestinal structure. In the jejunum, villus height was significantly greater in the CHM group than in the control and FHM groups (*p* = 0.002), while both CHM and FHM groups showed significantly reduced crypt depth compared to the control (*p* < 0.001). The V/C ratio was significantly elevated in both CHM and FHM groups, with the CHM group displaying the highest ratio (*p* < 0.001), indicating enhanced absorptive capacity. In the ileum, villus height was significantly higher in the CHM and FHM groups than in the control (*p* < 0.001). Crypt depth was also significantly increased in these groups (*p* = 0.004). However, no significant differences were detected in the V/C ratio among the three treatments (Figure S2).

### 3.4. Intestinal Barrier, Autophagy and Apoptosis

**Figure 1:**
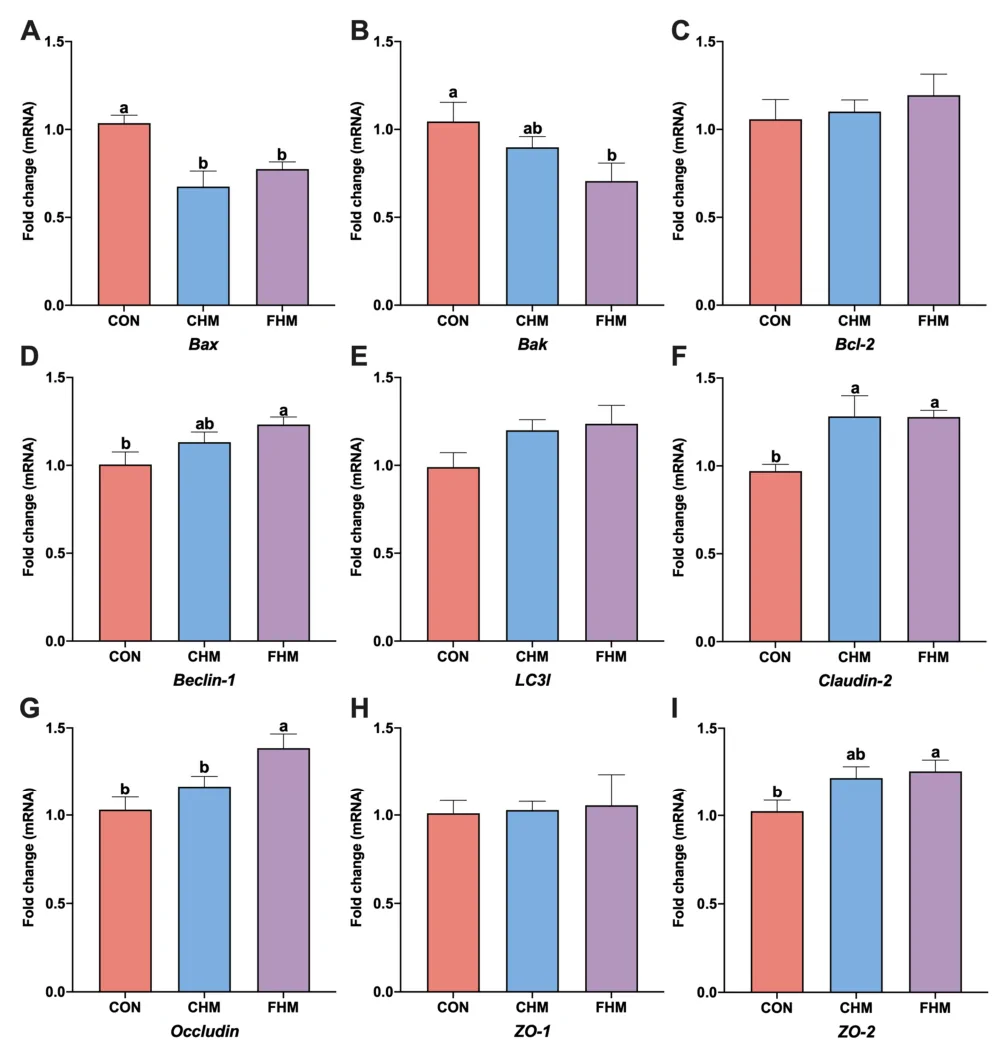
Effects of fermented Chinese herbal medicine on the relative mRNA expression of genes related to tight junction regulation, autophagy, and apoptosis in the ileum of aged laying hens. Relative mRNA expression of pro-apoptotic genes *Bax* (**A**) and *Bak* (**B**), and anti-apoptotic gene *Bcl-2* (**C**) in the ileum. Relative mRNA expression of autophagy-related genes *Beclin-1* (**D**), *LC3I* (**E**) in the ileum. Relative mRNA expression of tight junction genes *Claudin-2* (**F**), *Occludin* (**G**), *ZO-1* (**H**), *ZO-2* (**I**) in the ileum. Data are presented as means ± SEM (*n* = 6). *Bax* = Bcl-2-associated X protein; *Bak* = Bcl-2 homologous antagonist/killer; *Bcl-2* = B-cell lymphoma-2; *Beclin-1* = beclin-1 autophagy-related; *LC3I* = microtubule-associated proteins 1A/1B light chain 3 isoform I; *ZO-1* = zonula occludens-1; *ZO-2* = zonula occludens-2. CON = the control group; CHM = Chinese herbal medicine group; FHM = fermented Chinese herbal medicine group. Means without a common superscript letter differ significantly (*p* < 0.05).

The relative mRNA expression of genes related to apoptosis, autophagy, and tight junction regulation is shown in Figure 1. The mRNA expression levels of the pro-apoptotic genes Bcl-2-associated X protein (*Bax*) and Bcl-2 homologous antagonist/killer (*Bak*) were significantly lower in the FHM group than in the control group (*p* < 0.05). The mRNA expression of the autophagy-related gene *Beclin-1* was significantly higher in the FHM group than in the control group (*p* < 0.05). Among tight junction-related genes, the mRNA expression levels of *Claudin-2*, *Occludin*, and zonula occludens-2 (*ZO-2*) were significantly higher in the FHM group than in the control group (*p* < 0.05).

### 3.5. Serum Immunoglobulin and Intestinal Inflammatory Factors

Table 8 shows the effects of fermented Chinese herbs on serum immunoglobulin concentrations and intestinal inflammatory cytokines in laying hens. Regarding serum immunoglobulins, levels of IgA, IgG, and IgM were significantly increased in both the CHM and FHM groups compared with the control group (*p* < 0.05). Notably, the FHM group exhibited the highest IgG concentrations among all groups. In terms of intestinal inflammatory factors, the levels of IL-1β, IL-8, and TGF-β were significantly reduced in the CHM and FHM groups compared with the control group (*p* < 0.05), indicating changes in intestinal inflammatory and immunoregulatory responses. However, there was no significant difference in TNF-α levels among the groups (*p* > 0.05).

### 3.6. Cecal Microbial Diversity and Community Structures

**Figure 2:**
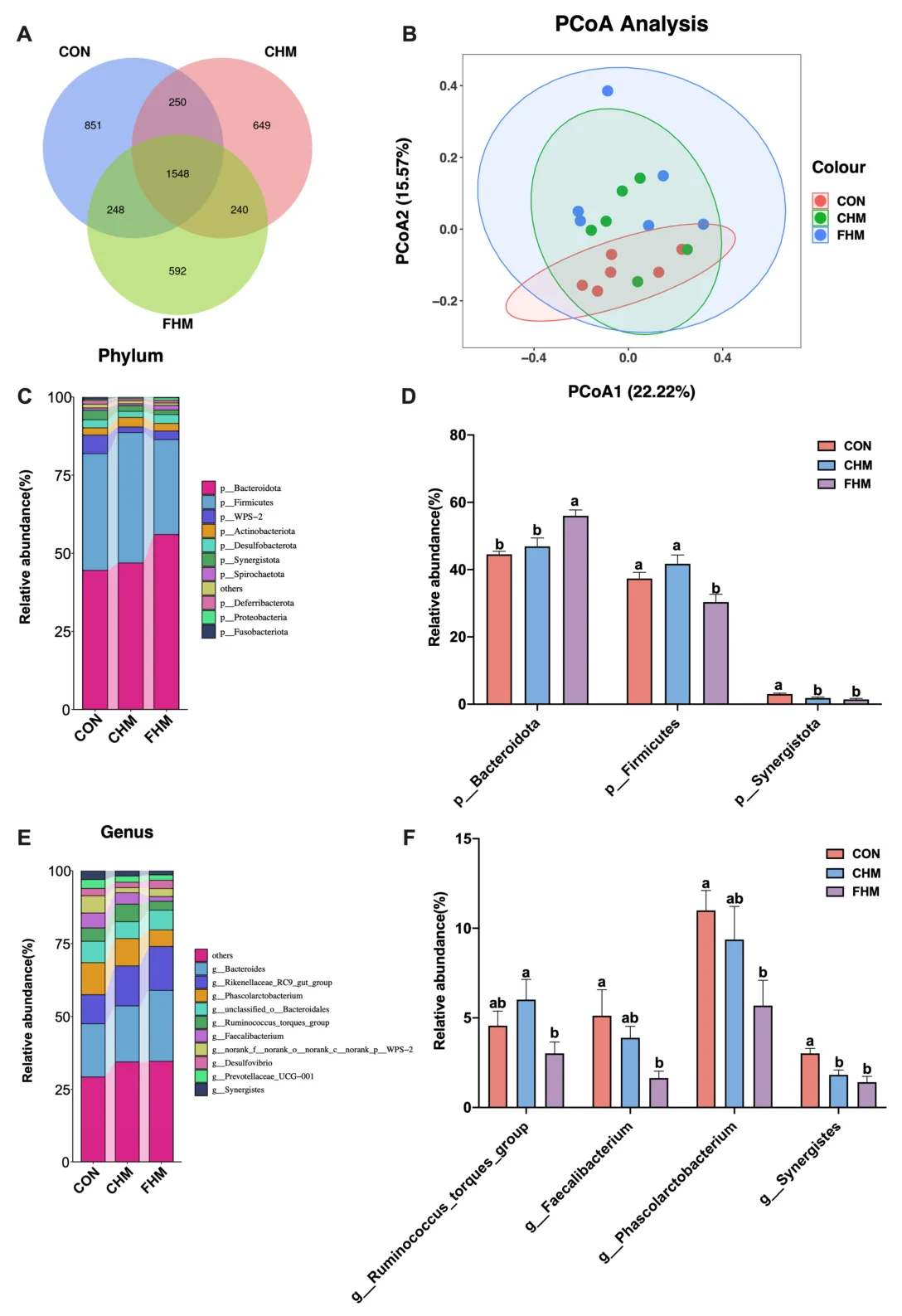
Effects of dietary fermented Chinese herbal medicine on cecal microbial diversity and community structure in aged laying hens. The Venn plot based on the total number of OTUs (**A**). PCoA analysis of all groups (**B**). Relative abundance of bacterial groups at the phylum level (**C**) and differential bacteria (**D**). Relative classification of bacterial groups at the genus level (**E**) and differential bacteria (**F**) in the cecum. CON = the control group; CHM = Chinese herbal medicine group; FHM = fermented Chinese herbal medicine group. Means without a common superscript letter differ significantly (*p* < 0.05).

16S rRNA sequencing analysis revealed that, at a 97% similarity threshold, the number of unique OTUs was highest in the control group (851), followed by the CHM group (649), and lowest in the FHM group (592) (Figure 2A). Principal coordinate analysis (PCoA) based on beta diversity demonstrated distinct clustering, indicating clear compositional differences in microbial communities among the three groups (Figure 2B). At the phylum level, the relative abundance of Bacteroidetes was significantly increased in the FHM group compared to both the control and CHM groups, whereas Firmicutes were more dominant in the control and CHM groups than in the FHM group. Additionally, the relative abundance of Synergistota was significantly higher in the control group than in the CHM and FHM groups (Figure 2C,D). At the genus level, the FHM group showed a marked reduction in the relative abundances of *Ruminococcus_torques_group*, *Faecalibacterium*, *Phascolarctobacterium*, and *Synergistes* compared to the control and CHM groups (Figure 2E,F).

### 3.7. Short-Chain Fatty Acids

**Figure 3:**
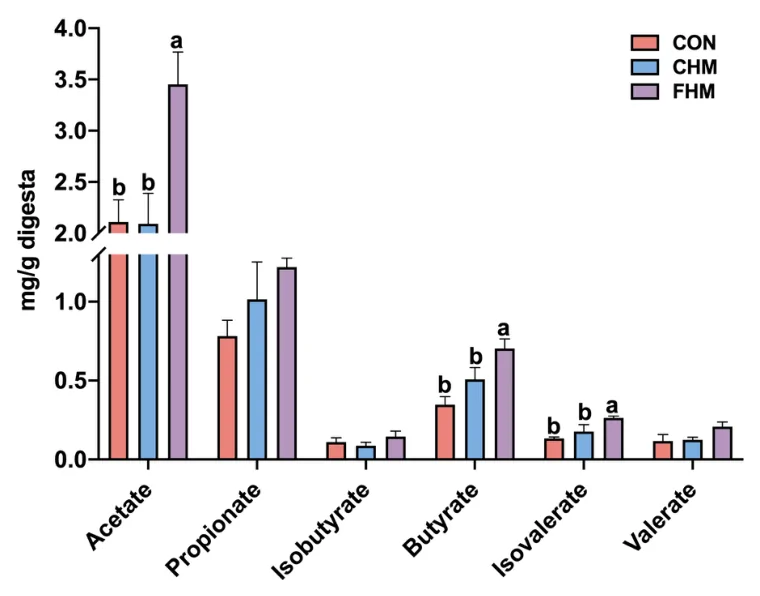
Effects of fermented Chinese herbal medicine on the short-chain fatty acids in aged laying hens. Data are presented as means ± SEM (*n* = 6). CON = the control group; CHM = Chinese herbal medicine group; FHM = fermented Chinese herbal medicine group. Means without a common superscript letter differ significantly (*p* < 0.05).

Analysis of cecal short-chain fatty acids demonstrated that the concentrations of acetate, butyrate, and isovalerate were significantly higher in the FHM group compared with both the control and CHM groups (*p* < 0.05) (Figure 3). In contrast, no significant difference was detected between the CHM and control groups (*p* > 0.05).

## 4. Discussion

Egg production and egg quality are key parameters for evaluating the performance of laying hens. Previous studies have demonstrated that supplementation with CHM or FHM can enhance poultry productivity and improve Haugh unit scores [28,29]. Previous studies have reported favorable effects of fermented herbal supplements on egg production and egg quality [28,30,31]. Fermentation may modify the nutritional composition and bioactive constituents of herbal products [32,33]. However, because the CHM and FHM treatments in the present study differed in both composition and inclusion level, the contribution of fermentation to the observed effects cannot be determined independently. It has also been reported that Fufang Zhenzhu Tiao Zhi (FTZ) possesses anti-inflammatory and antioxidant properties and can delay intestinal aging by modulating intestinal morphology and the microbiota [34]. In the present study, FHM supplementation was associated with improvements in selected intestinal health-related indicators and production traits in late-phase laying hens. However, these findings do not establish an anti-aging effect.

Because oxidative stress is a major feature of intestinal aging, the effects of FHM on antioxidant status may partly explain its beneficial effects on production performance. During intestinal aging, diminished defense capacity and oxidative stress induced by reactive oxygen species (ROS) can impair the normal function of the intestinal epithelium through multiple pathways, ultimately contributing to various intestinal disorders [35,36]. Recently, targeting cellular stress responses and ROS has emerged as a potential therapeutic strategy for treating intestinal diseases [36]. Although serum GSH-Px activity was lower in the CHM and FHM groups, both treatments significantly reduced serum MDA concentrations and increased SOD activity, suggesting that dietary supplementation alleviated oxidative stress through coordinated regulation of multiple antioxidant defense pathways rather than by uniformly enhancing all antioxidant enzymes. The changes in ovarian GSH-Px and CAT activities further suggest tissue-dependent antioxidant responses to dietary herbal supplementation. Previous studies have shown that *Codonopsis pilosula* enhances intestinal SOD levels and alleviates inflammatory damage in the gut [37]. In addition, Glycyrrhizae radix (GR), a common ingredient in all formulas, has been reported to suppress intestinal ROS production and prolong lifespan in nematode models [38], consistent with our findings. These findings suggest that herbal supplementation may modulate antioxidant responses in late-phase laying hens, although its effects on biological aging remain unknown.

In addition to oxidative stress, age-related structural deterioration of the intestinal mucosa can further compromise nutrient digestion and absorption [39]. Age-related impairment of the intestinal barrier is a common pathophysiological feature of aging [40]. It is well known that aging leads to structural and functional deterioration of the gastrointestinal tract, including mucosal atrophy [41], as well as reduced digestive efficiency and impaired nutrient absorption [42]. In the present study, dietary CHM and FHM supplementation improved selected intestinal morphological parameters, including villus height, crypt depth, and the villus-to-crypt ratio, depending on the intestinal segment examined. These findings suggest potential benefits for intestinal structure in late-phase laying hens. However, whether these morphological changes translate into improved nutrient absorption or attenuation of age-related intestinal deterioration requires further investigation [43]. *Poria cocos* and *Cornus officinalis* may contribute to the observed changes in intestinal health-related indicators in late-phase laying hens [17,18]. The expression levels of tight junction proteins, as well as genes related to autophagy and apoptosis, play crucial roles in maintaining intestinal barrier integrity. Consistent with these findings, FHM supplementation altered the mRNA expression of several tight junction-related genes and reduced the mRNA expression of *Bax* and *Bak*. These findings suggest potential changes in intestinal barrier-related and apoptosis-related signaling, although corresponding protein expression and functional changes were not directly evaluated. Similar findings were reported by Che et al. [44].

Intestinal barrier function and inflammatory signalling are closely related, and tight-junction components participate in epithelial regulation [45,46]. In laying hens, experimentally induced intestinal inflammation has been associated with hepatic inflammation and reduced expression of yolk precursor-related genes. Intestinal inflammation may impair nutrient absorption and compromise hepatic yolk precursor synthesis, potentially affecting ovarian follicular development and egg production, partly through endotoxin-associated hepatic inflammation [47]. In our study, dietary supplementation with CHM significantly increased serum IgA levels and reduced the pro-inflammatory cytokine IL-1β. These findings are consistent with those reported by Song et al. [48,49]. Unlike the pro-inflammatory cytokines IL-1β and IL-8, TGF-β plays an important role in immune regulation and intestinal mucosal homeostasis. Therefore, changes in TGF-β levels should be interpreted as alterations in immunoregulatory responses rather than direct evidence of reduced inflammation. Furthermore, Citri reticulatae Pericarpium (Chenpi), *Cornus officinalis*, and *Poria* have been shown to significantly reduce inflammatory markers such as IL-8 and TNF-α [50]. Based on this evidence, we hypothesize that FHM may improve egg production performance in laying hens by modulating immune responses [45,46,51]. These findings suggest that FHM supplementation may influence selected immune parameters in late-phase laying hens, although its effects on intestinal inflammation and aging require further investigation.

Because the gut microbiota is closely involved in intestinal barrier integrity and immune homeostasis, the microbial changes observed in the present study may represent another mechanism underlying the effects of FHM. Previous studies have shown that CHM can help maintain the stability and normal physiological function of the intestinal barrier by increasing the relative abundance of beneficial bacteria [44]. Thus, CHM may have the potential to support intestinal health through modulation of the gut microbiota [52]. In particular, members of the order Bacteroidales are known to contribute to SCFA production [53], nutrient utilization, and immune function [54]. Previous studies have shown that the recovery of gut microbial communities, including Lachnospirales and Oscillospirales, following transient microbiota disruption can influence *Salmonella* Typhimurium colonization dynamics and the selection of virulence-associated genotypes [55]. These findings highlight the potential role of gut microbiota in host–pathogen interactions, although whether similar mechanisms contribute to intestinal health in aged laying hens requires further investigation. Moreover, microbiota-derived SCFAs can enhance intestinal nutrient absorption [56] and may play a role in mitigating the age-related decline in egg quality observed in aged laying hens [57,58]. These findings suggest that FHM may indirectly improve production performance in aging hens by promoting microbial homeostasis, increasing the generation of beneficial microbial metabolites, and regulating nutrient absorption and immune responses. Although changes in lactic acid bacteria and *Bacillus * populations were monitored during fermentation, the viability of individual inoculated microorganisms after refrigerated storage and at the time of feeding was not evaluated. Therefore, the observed effects cannot be attributed specifically to the presence of viable inoculated microorganisms. Future studies may identify the bioactive components of the herbal medicine used in this study through mass spectrometry and apply network pharmacology approaches to investigate their potential targets.

## 5. Conclusions

In summary, dietary supplementation with FHM may improve selected production traits, egg quality, and intestinal health indicators in aged laying hens. However, overall egg production rate and FCR did not differ significantly among treatments over the entire experimental period, and FHM was not consistently superior to CHM across all measured outcomes. These effects were associated with changes in antioxidant status, immune-related parameters, intestinal morphology, and gut microbiota. Further studies are needed to elucidate the specific mechanisms underlying these effects. These findings suggest that FHM may serve as a potential antibiotic-free nutritional strategy for supporting intestinal health and selected production traits in aged laying hens.

## Figures and Tables

**Table 1:** Composition of the Chinese herbal formula.

Chinese Herbal Medicine	Proportion in Herbal Mixture (%)	CHM Contribution (g/kg Diet)
Dang Gui (*Angelica sinensis*)	14.00	0.672
Huang Qi (*Astragalus membranaceus*)	14.00	0.672
Xu Duan (*Dipsacus asper*)	9.00	0.432
Fu Ling (*Poria cocos*)	25.00	1.200
Shan Zhu Yu (*Cornus officinalis*)	15.00	0.720
Chen Pi (*Citrus reticulata*)	20.00	0.960
Gan Cao (*Glycyrrhiza uralensis*)	3.00	0.144
Total	100.00	4.800

**Table 2:** Composition and nutrient levels of the experimental diets (air-dry basis).

Item	Control	CHM	FHM
Ingredient composition (%)			
Corn	36.00	35.83	35.28
Wheat	35.00	34.83	34.30
Soybean meal	17.30	17.22	16.95
Calcium hydrogen phosphate	0.80	0.80	0.78
Stone powder	9.40	9.35	9.21
Premix ^1^	1.50	1.49	1.48
Chinese herbal feed	-	0.48	2.00
Total	100.00	100.00	100.00
Nutrient composition			
Metabolizable energy ^2^ (kcal/kg)	2630	2630	2630
Crude protein (%)	14.85	14.80	14.69
Calcium (%)	3.56	3.51	3.50
Total phosphorus (%)	0.51	0.51	0.53
Lysine (%)	0.78	0.78	0.78
Methionine (%)	0.38	0.38	0.38
Methionine + Cysteine (%)	0.63	0.63	0.63

^1^ Premix supplied per kilogram of diet: VA: 10,000 IU, VD_3_: 3500 IU, VE: 25 mg, VK_3_: 3 mg, VB_1_: 2 mg, VB_2_: 5 mg, VB_6_: 3 mg, VB_12_: 0.02 mg, Niacin: 35 mg, d-Calcium pantothenate: 15 mg, Folic acid: 1 mg, Biotin: 0.1 mg, Choline: 200 mg, Copper: 8 mg, Iron: 40 mg, Manganese: 100 mg, Zinc: 80 mg, Iodine: 1.2 mg, Selenium: 0.3 mg, L-Lysine monohydrochloride: 2 g, DL-Methionine: 1.5 g, Salt: 3 g. ^2^ Metabolizable energy was a calculated value, while the others were measured values.

**Table 3:** Primer sets for quantitative real-time PCR.

Gene	Primer	Primer Sequence (5′—3′)	Size (bp)
*Claudin-2*	Forward	CTCAGCCCTCCATCAAACA	82
	Reverse	TGCTGCTGCTACACGTAT	
*Occludin*	Forward	GCAGATGTCCAGCGGTTACTAC	176
	Reverse	CGAAGAAGCAGATGAGGCAGAG	
*ZO-1*	Forward	CAAGGAGCCATTCCAGAGGG	125
	Reverse	CCACACATTACCAAGGGGCT	
*ZO-2*	Forward	TCACCTTCTTCTTCTTCCCAATCT	178
	Reverse	GCAACCTTTCCGTCCATCTC	
*Bax*	Forward	TCCTCATCGCCATGCTCAT	69
	Reverse	CCTTGGTCTGGAAGCAGAAGA	
*Bak*	Forward	ATGGATGCCTGTCTGTCCTGTTC	106
	Reverse	GCAGAGCAGTCCAAAGACACTGA	
*Bcl-2*	Forward	GATCGTCGCCTTCTTCGAGT	217
	Reverse	GGCCTCATACTGTTGCCGTA	
*Beclin-1*	Forward	CGTATGGCAACCACTCGTATT	97
	Reverse	TTATTGTCCCAGAAGAACCTCAG	
*LC3I*	Forward	TTACACCCATATCAGATTCTTG	143
	Reverse	ATTCCAACCTGTCCCTCA	
*β-actin*	Forward	TGCGTGACATCAAGGAGAAG	300
	Reverse	TGCCAGGGTACATTGTGGTA	

*ZO-1* = zonula occludens-1; *ZO-2* = zonula occludens-2; *Bax* = Bcl-2-associated X protein; *Bak* = Bcl-2 homologous antagonist/killer; *Bcl-2* = B-cell lymphoma-2.

**Table 4:** Effect of fermented Chinese herbs on laying performance of aged laying hens.

Item	Diet			SEM	*p*-Value
	Control	CHM	FHM		
1–4 W					
Daily egg production, %	85.67	86.23	86.43	0.464	0.852
Average egg weight, g	60.47 ^b^	61.13 ^ab^	61.62 ^a^	0.203	0.035
ADFI, g/bird/d	130.15 ^a^	117.96 ^b^	110.41 ^c^	2.690	0.001
FCR, g/g	2.42 ^a^	2.21 ^ab^	2.16 ^b^	0.047	0.045
5–8 W					
Daily egg production, %	88.41	89.40	89.93	0.373	0.262
Average egg weight, g	61.35 ^b^	61.89 ^b^	62.60 ^a^	0.202	0.009
ADFI, g/bird/d	133.43 ^a^	115.28 ^c^	121.70 ^b^	2.486	<0.001
FCR, g/g	2.40	2.31	2.25	0.044	0.400
9–12 W					
Daily egg production, %	87.33 ^b^	89.35 ^a^	89.60 ^a^	0.356	0.002
Average egg weight, g	61.93 ^c^	62.60 ^b^	63.38 ^a^	0.216	<0.001
ADFI, g/bird/d	133.43 ^a^	122.47 ^b^	121.40 ^b^	1.858	<0.001
FCR, g/g	2.37	2.35	2.27	0.036	0.479
1–12 W					
Daily egg production, %	87.49	88.09	88.55	0.278	0.382
Average egg weight, g	61.27 ^b^	61.53 ^b^	62.52 ^a^	0.201	0.001
ADFI, g/bird/d	132.00 ^a^	118.55 ^b^	117.77 ^b^	2.255	0.004
FCR, g/g	2.46	2.19	2.13	0.058	0.128

CHM = Chinese herbal medicine; FHM = fermented Chinese herbal medicine; ADFI = average daily feed intake; FCR = feed conversion ratio. Means with different superscripts within the same row differ significantly (*p* < 0.05).

**Table 5:** Effect of fermented Chinese herbs on egg quality of aged laying hens.

Item	Diet			SEM	*p*-Value
	Control	CHM	FHM		
Eggshell color	28.85	27.75	27.24	0.314	0.096
Egg shape index	1.30	1.29	1.30	0.003	0.728
Eggshell thickness, mm	0.31	0.32	0.32	0.002	0.228
Eggshell strength, N	39.57	41.41	41.74	0.429	0.066
Yolk color	6.68 ^a^	6.01 ^b^	6.96 ^a^	0.147	0.007
Yolk ratio	0.29 ^a^	0.28 ^b^	0.28 ^b^	0.002	0.030
Haugh unit	80.70 ^b^	83.55 ^a^	84.40 ^a^	0.594	0.009

CHM = Chinese herbal medicine; FHM = fermented Chinese herbal medicine. Means with different superscripts within the same row differ significantly (*p* < 0.05).

**Table 6:** Effects of fermented Chinese herbs on antioxidant properties of aged laying hens.

Item		Diet			SEM	*p*-Value
		Control	CHM	FHM		
Serum	GSH-Px, U/mL	1665.45 ^a^	594.30 ^c^	843.04 ^b^	152.254	<0.001
	SOD, U/mL	1313.18 ^c^	1983.62 ^b^	2623.72 ^a^	186.014	0.002
	MDA, nmol/mL	6.11 ^a^	4.10 ^b^	4.04 ^b^	0.338	0.002
	CAT, U/mL	0.55 ^b^	2.26 ^a^	1.02 ^b^	0.278	0.004
Liver	GSH-Px, U/mg prot	33.17	32.19	30.43	1.862	0.857
	SOD, U/mg prot	886.67 ^c^	1148.62 ^b^	1383.45 ^a^	75.269	<0.001
	MDA, nmol/mg prot	0.59	0.74	0.63	0.030	0.091
	CAT, U/mg prot	33.34	36.43	40.73	2.564	0.532
Ovary	GSH-Px, U/mg prot	26.40 ^b^	36.45 ^a^	34.80 ^a^	1.609	0.002
	SOD, U/mg prot	337.08 ^b^	426.76 ^a^	387.20 ^ab^	14.039	0.008
	MDA, nmol/mg prot	2.37 ^a^	0.74 ^b^	0.96 ^b^	0.274	0.002
	CAT, U/mg prot	3.35 ^b^	3.68 ^b^	6.88 ^a^	0.603	0.011

CHM = Chinese herbal medicine; FHM = fermented Chinese herbal medicine; SOD = superoxide dismutase; GSH-Px = glutathione peroxidase; CAT = catalase; MDA = malondialdehyde. Means with different superscripts within the same row differ significantly (*p* < 0.05).

**Table 7:** Effects of fermented Chinese herbs on intestinal morphology of aged laying hens.

Item		Diet			SEM	*p*-Value
		Control	CHM	FHM		
Duodenum	V, μm	1429.15	1481.39	1508.00	41.951	0.774
	C, μm	257.26 ^a^	202.52 ^b^	184.20 ^b^	12.833	0.033
	V/C	5.56 ^b^	7.33 ^ab^	8.54 ^a^	0.496	0.026
Jejunum	V, μm	777.96 ^b^	1126.92 ^a^	910.81 ^b^	49.800	0.002
	C, μm	295.82 ^a^	169.02 ^b^	156.68 ^b^	19.715	<0.001
	V/C	2.64 ^c^	6.67 ^a^	5.82 ^b^	0.532	<0.001
Ileum	V, μm	613.03 ^b^	930.80 ^a^	917.12 ^a^	47.353	<0.001
	C, μm	129.70 ^b^	180.91 ^a^	169.05 ^a^	7.853	0.004
	V/C	4.85	5.18	5.43	0.218	0.594

CHM = Chinese herbal medicine; FHM = fermented Chinese herbal medicine; V = villus height; C = crypt depth; V/C = the villus height-to-crypt depth ratio. Means with different superscripts within the same row differ significantly (*p* < 0.05).

**Table 8:** Effects of fermented Chinese herbs on serum immunoglobulin and intestinal inflammatory factors of aged laying hens.

Item		Diet			SEM	*p*-Value
		Control	CHM	FHM		
Serum	IgA, μg/mL	252.12 ^b^	360.84 ^a^	344.22 ^a^	15.736	<0.001
	IgG, μg/mL	1796.09 ^c^	2207.06 ^b^	2439.13 ^a^	89.98	0.001
	IgM, μg/mL	685.61 ^c^	879.95 ^a^	835.97 ^b^	29.842	<0.001
Ileum	IL-1β, pg/mL	3659.00 ^a^	1909.43 ^b^	1704.83 ^b^	284.645	<0.001
	IL-8, pg/mL	712.32 ^a^	465.33 ^b^	455.67 ^b^	40.338	0.003
	TGF-β, pg/mL	461.44 ^a^	260.91 ^b^	236.61 ^b^	32.765	<0.001
	TNF-α, pg/mL	1150.93	1160.73	1271.68	68.629	0.765

CHM = Chinese herbal medicine; FHM = fermented Chinese herbal medicine; IgA = immunoglobulin A; IgG = immunoglobulin G; IgM = immunoglobulin M; IL-1β = interleukin-1 beta; IL-8 = interleukin-8; TNF-α = tumor necrosis factor-alpha; TGF-β = transforming growth factor-beta. Means with different superscripts within the same row differ significantly (*p* < 0.05).

## Data Availability

The raw reads of 16S rRNA sequencing are available at NCBI project PRJNA1538980.

## Supplementary Materials

The following supporting information can be downloaded at: https://www.mdpi.com/article/10.3390/ani16213510/s1, Figure S1. Changes in lactic acid bacteria, *Bacillus subtilis* and pH value during the fermentation process. Figure S2. Effects of fermented Chinese herbs on intestinal morphology of aged laying hens. Table S1. Detection of basic chemical components before and after fermentation.
