# Supplementary material for: Effects of Fermented and Unfermented Chinese Herbal Formulations on Laying Performance, Intestinal Health and Cecal Microbiota in Late-Phase Laying Hens
Source: Animals (Basel). 2026 Nov 6;16(21):3510. doi: 10.3390/ani16213510 (PMC13761426; doi:10.3390/ani16213510)
Supplement: Supplementary file 1 [file animals-16-03510-s001.zip › animals-4669344-supplementary.pdf]

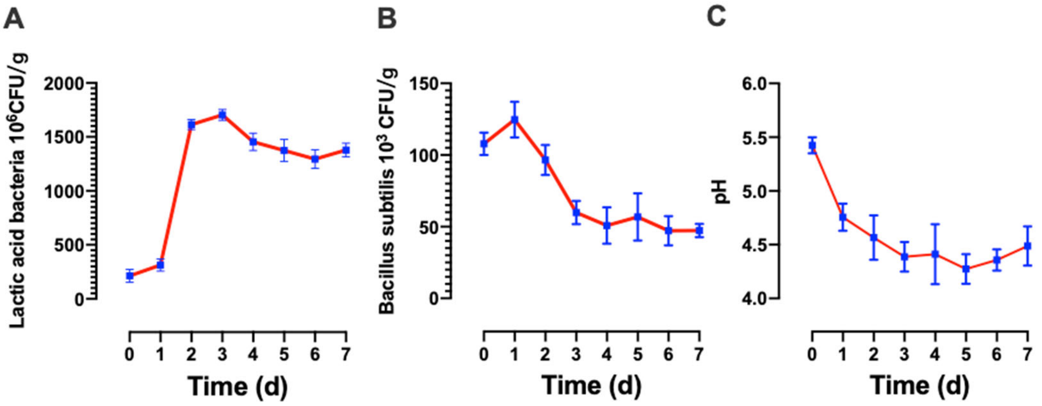

Figure S1. Changes in lactic acid bacteria, *Bacillus subtilis* and pH value during the fermentation process (**A**) Changes in the number of *Lactobacillus* species; (**B**) Changes in the number of *Bacillus subtilis* species; (**C**) Changes in pH

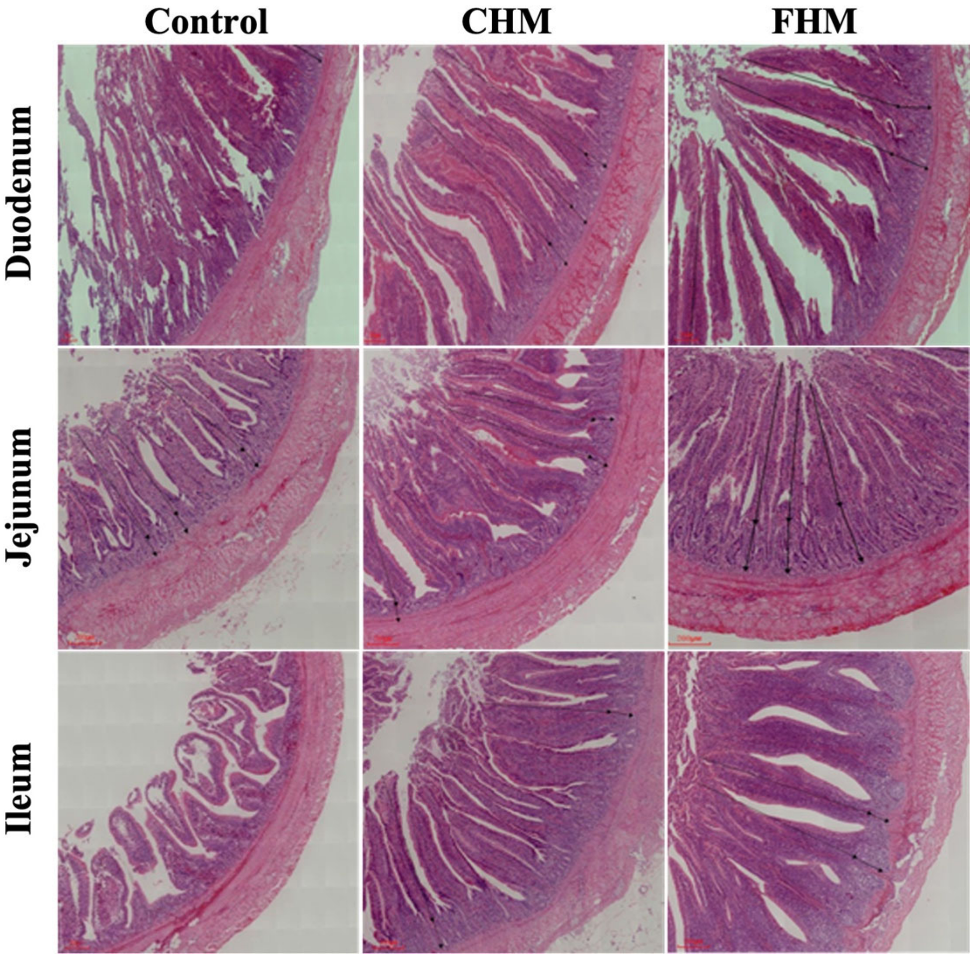

Figure S2. Effects of fermented Chinese herbs on intestinal morphology of aged laying hens

Table S1. Detection of Basic Chemical Components Before and After Fermentation

Item	Day		SEM	*P*-valve
	0 d	7 d		
Crude protein, %	13.81	14.29	0.339	0.230
Ether extract, %	4.26[b]	5.10[a]	0.078	0.005
Dry matter, %	68.03	62.91	2.732	0.134
Crude ash, %	6.94	8.65	0.515	0.078
NDF, %	34.96	33.82	1.160	0.383
ADF, %	23.28	21.72	1.160	0.250
Lactic acid, g/kg	10.70[b]	22.26[a]	0.415	<0.001

Abbreviation: NDF, neutral detergent fiber. ADF, acid detergent fiber.
